# Localization of *Kif1c* mRNA to cell protrusions dictates binding partner specificity of the encoded protein

**DOI:** 10.1101/gad.350320.122

**Published:** 2023-03-01

**Authors:** Megan L. Norris, Joshua T. Mendell

**Affiliations:** 1 Department of Molecular Biology, University of Texas Southwestern Medical Center, Dallas, Texas 75390, USA;; 2 Harold C. Simmons Comprehensive Cancer Center, University of Texas Southwestern Medical Center, Dallas, Texas 75390, USA;; 3 Hamon Center for Regenerative Science and Medicine, University of Texas Southwestern Medical Center, Dallas, Texas 75390, USA;; 4 Howard Hughes Medical Institute, University of Texas Southwestern Medical Center, Dallas, Texas 75390, USA

**Keywords:** RNA localization, RNA transport, kinesin, post-transcriptional regulation, protein–protein interactions

## Abstract

Subcellular localization of messenger RNA (mRNA) is a widespread phenomenon that can impact the regulation and function of the encoded protein. In nonneuronal cells, specific mRNAs localize to cell protrusions, and proper mRNA localization is required for cell migration. However, the mechanisms by which mRNA localization regulates protein function in this setting remain unclear. Here, we examined the functional consequences of localization of the mRNA encoding KIF1C. KIF1C is a kinesin motor protein required for cell migration and mRNA trafficking, including trafficking of its own mRNA. We show that *Kif1c* mRNA localization does not regulate KIF1C's protein abundance, distribution, or ability to traffic other mRNAs. Conversely, *Kif1c* mRNA localization to protrusions is required for directed cell migration. We used mass spectrometry to identify binding partners of endogenous KIF1C, which revealed dramatic dysregulation of the number and identity of KIF1C interactors in response to *Kif1c* mRNA mislocalization. These results therefore uncovered a mechanistic connection between mRNA localization to cell protrusions and the specificity of protein–protein interactions. We anticipate that this mechanism is not limited to *Kif1c* and is likely to be a general principle that impacts the functions of proteins encoded by protrusion-enriched mRNAs in nonneuronal cells.

## Abstract (precis)

In this study, Norris and Mendell sought to understand the mechanisms by which mRNA localization regulates protein function in nonneuronal cells during cell migration. They examined the functional consequences of localization of the mRNA encoding KIF1C, a kinesin motor protein required for cell migration and mRNA trafficking, and show that *Kif1c* mRNA localization does not regulate KIF1C's protein abundance, distribution, or ability to traffic other mRNAs and provide insight into a mechanistic connection between mRNA localization to cell protrusions and the specificity of protein–protein interactions.

Subcellular localization of mRNA is a widely occurring form of post-transcriptional regulation that can tune protein output in space and time. While mRNA localization has been most extensively studied in large, highly asymmetrical systems, such as oocytes and neurons, it also occurs in smaller cells including both mesenchymal and epithelial cell types (for review, see Engel et al. 2020; Gasparski et al. 2022). In nonneuronal migratory cells, disruption of mRNA localization to cellular protrusions routinely leads to defective cell migration and has been shown to affect processes in vivo including cancer cell invasion and blood vessel morphogenesis (Wang et al. 2017; Chrisafis et al. 2020; Costa et al. 2020; Moissoglu et al. 2020). It was predicted that mechanistic principles of mRNA localization in nonneuronal cells would be similar to those of neurons, but recent work has challenged that assumption. In neuronal systems, *cis*-elements in localized mRNAs are bound by RNA binding proteins, which are then carried by molecular motors into neurites and docked using the same or different proteins (Das et al. 2019). The mRNAs are generally translationally silent during transport and undergo local translation at their final site in the neurite, often in response to a stimulus. In contrast, global mRNA and protein localization patterns are not correlated in protrusions of nonneuronal cells (Mardakheh et al. 2015). Furthermore, at least some localized mRNAs are translated en route to nonneuronal cell protrusions, only to be translationally silenced at their destination (Moissoglu et al. 2019). Thus, the functional impact of mRNA localization to protrusions in nonneuronal cells cannot be directly inferred from principles established in neurons, necessitating further empirical characterization of the molecular and phenotypic consequences of mRNA mislocalization in nonneuronal cell types.

Work to date has revealed at least two classes of protrusion-localized RNAs in nonneuronal cells: those that require the tumor suppressor APC to localize (“APC-dependent”) and those that do not (“APC-independent”) (Mili et al. 2008; Wang et al. 2017). While important aspects of the localization mechanisms for both groups remain to be elucidated, key insights support the hypothesis that each group uses its own set of general principles for localization. For example, multiple APC-dependent mRNAs have been definitively shown or predicted to have guanine and adenine (GA)-rich *cis*-elements that mark them for trafficking (Chrisafis et al. 2020; Costa et al. 2020; Moissoglu et al. 2020; Arora et al. 2022). Many, if not all, APC-dependent mRNAs also require the kinesin KIF1C for trafficking (Pichon et al. 2021). Conversely, many APC-independent mRNAs, which include a large number of ribosomal protein mRNAs, use LARP family members to localize (Wang et al. 2017; Dermit et al. 2020; Goering et al. 2022). Intriguingly, these two classes of localized mRNAs seem to populate different types or stages of protrusions (Wang et al. 2017). A glimpse into the molecular consequences of localizing APC-dependent mRNAs has been provided by detailed studies of *Rab13*. Localization of *Rab13* mRNA to protrusions does not affect RAB13 protein abundance or distribution but regulates cotranslational loading of a key interacting partner (Moissoglu et al. 2020). Without this regulated loading, cells with mislocalized *Rab13* mRNA phenocopy RAB13 knockdown cells, exhibiting a migration defect. However, whether mRNA localization to protrusions commonly regulates protein–protein interactions or whether this mechanism is unique to *Rab13* remains to be determined.

Another representative APC-dependent mRNA is *Kif1c*, which encodes a kinesin-3 motor protein (Dorner et al. 1998). *Kif1c* is one of the most commonly localized mRNAs across cell types and has been observed in protrusions in diverse cell lines including HeLa, mouse fibroblasts (NIH3T3), and human umbilical vein endothelial cells (HUVECs) (Wang et al. 2017; Chouaib et al. 2020; Costa et al. 2020). Previous work has determined that the *Kif1c* 3′ UTR is sufficient to drive localization of reporter mRNAs and is predicted to include GA elements, similar to other APC-dependent mRNAs (Chrisafis et al. 2020; Costa et al. 2020; Moissoglu et al. 2020). KIF1C protein is required for directed cell migration and proper transport of α5β1 integrin to focal adhesions, specifically in the rear of the cell (Theisen et al. 2012). As described above, KIF1C also interacts with APC to traffic its own mRNA, as well as other APC-dependent mRNAs (Pichon et al. 2021). These studies paint a picture in which *Kif1c* mRNA localization is a carefully orchestrated process shared across organisms and cell types and predicts that *Kif1c* mRNA localization and protein function may be intertwined. Nevertheless, it is unclear whether localization of the *Kif1c* mRNA is required for proper KIF1C-mediated mRNA trafficking or cell migration.

Here, we tease apart the multiple functions of KIF1C and uncover their specific dependencies on mRNA localization. We found that *Kif1c* mRNA localization is dispensable for trafficking other APC-dependent mRNAs and has no effect on KIF1C protein abundance or distribution. Conversely, mislocalization of *Kif1c* mRNA leads to a broad reprogramming of KIF1C protein–protein interactions and results in defective cell migration. Thus, *Kif1c* mRNA localization appears to be critical for the establishment of distinct functional pools of KIF1C protein that are produced in different cellular compartments, thereby providing access to distinct sets of cargoes.

## Results

### Identification of *Kif1c* as a model localized RNA

**Figure 1.**
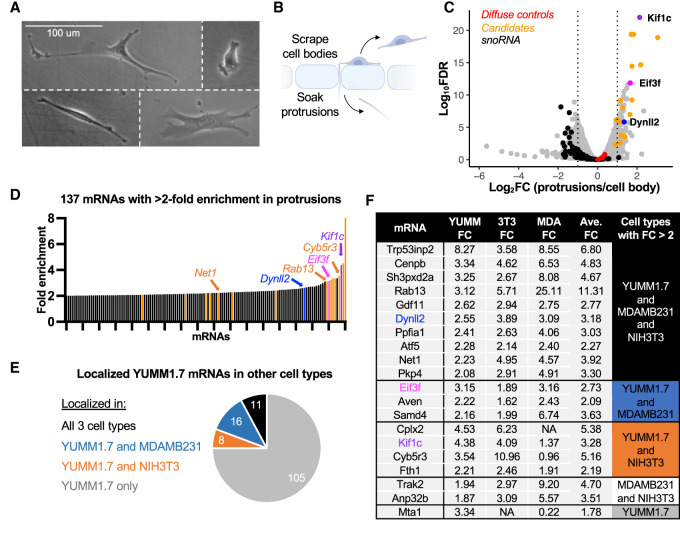
Identification and prioritization of mRNAs localized to protrusions in melanoma cells. (*A*) YUMM1.7 cells are a morphologically diverse and migratory cell type. (*B*) YUMM1.7 cells were fractionated into protrusions and cell bodies using microporous membranes. (*C*) RNA-seq results for fractionated YUMM1.7 cells. Control mRNAs such as *Ppia*, *Ywhaz*, *RhoA*, and *Arpc3* are uniformly distributed between protrusions and cell bodies (red dots), while nucleolar RNAs are depleted from protrusions (black). (*D*) Protrusion enrichment values for the top 137 localized mRNAs in YUMM1.7 cells. In *C* and *D*, *Dynll2*, *Eif3f*, *Kif1c*, and other candidate genes are denoted in color. (*E*) Thirty-five mRNAs that localize in melanoma cells are also localized in breast cancer cells (blue), embryonic fibroblasts (orange), or all three cell types (black). (*F*) Candidate mRNAs and their protrusion enrichment patterns in three nonneuronal cell lines. The table is arranged based on the candidates’ enrichments across three, two, or one cell type(s).

To gain mechanistic insight into the manner by which mRNA localization to protrusions affects cellular behavior, we focused on the mouse melanoma cell line YUMM1.7. This cell line is phenotypically rich, amenable to genetic manipulation, and genetically engineered to resemble human melanoma through activated *Braf* and deactivated *Pten* and *Cdkn2a* (Fig. 1A; Meeth et al. 2016). To identify RNAs that are localized to protrusions in these melanoma cells, we carried out a candidate prioritization pipeline consisting of four phases: identification, comparative analysis, phenotypic screening, and *cis*-element identification.

#### Phase 1: identification

Cells were plated on microporous membranes coated on the underside with fibronectin. Protrusions, but not cell bodies, are able to fit through the 1-μm pores (Fig. 1B). Cell bodies were collected from the top of the membrane by scraping, followed by collection of protrusions by soaking the resulting membranes in lysis buffer. RNA was isolated from protrusion and cell body samples and subjected to RNA sequencing (RNA-seq) (Supplemental Table S1). After excluding lowly expressed mRNAs (TPM < 1 in the cell bodies), we identified 137 mRNAs that were twofold or more enriched in protrusions compared with the cell body (Fig. 1C,D). mRNAs with well-studied subcellular localization patterns behaved as expected in these data. For example, *Arpc3* and *RhoA* mRNAs were uniformly distributed (near 0 in Fig. 1C, red dots), while *Rab13*, *Cyb5r3* and *Net1*, which are known to be localized to protrusions in other nonneuronal cells, were enriched in protrusions (Fig. 1D).

#### Phase 2: comparative analysis

We narrowed down our list of candidate mRNAs in two ways. First, we selected nine of the mRNAs that were most enriched in YUMM1.7 cells. Second, we assessed which mRNAs are protrusion enriched in other nonneuronal cell types (fibroblasts and/or breast cancer) (Jakobsen et al. 2013; Mardakheh et al. 2015; Wang et al. 2017). While most of the localized mRNAs we identified were specific to YUMM1.7 cells (105 out of 137), many were localized in one or both of the other cell types (Fig. 1E; Supplemental Table S1). We selected 11 of these shared, localized mRNAs with the reasoning that, although the absolute enrichment of these mRNAs may be more moderate, they may have a higher chance of being biologically relevant due to their shared enrichment across cell types. In this way, we chose 20 candidate mRNAs that were particularly strongly and/or commonly enriched in protrusions (Fig. 1F).

#### Phase 3: phenotypic screening

**Figure 2.**
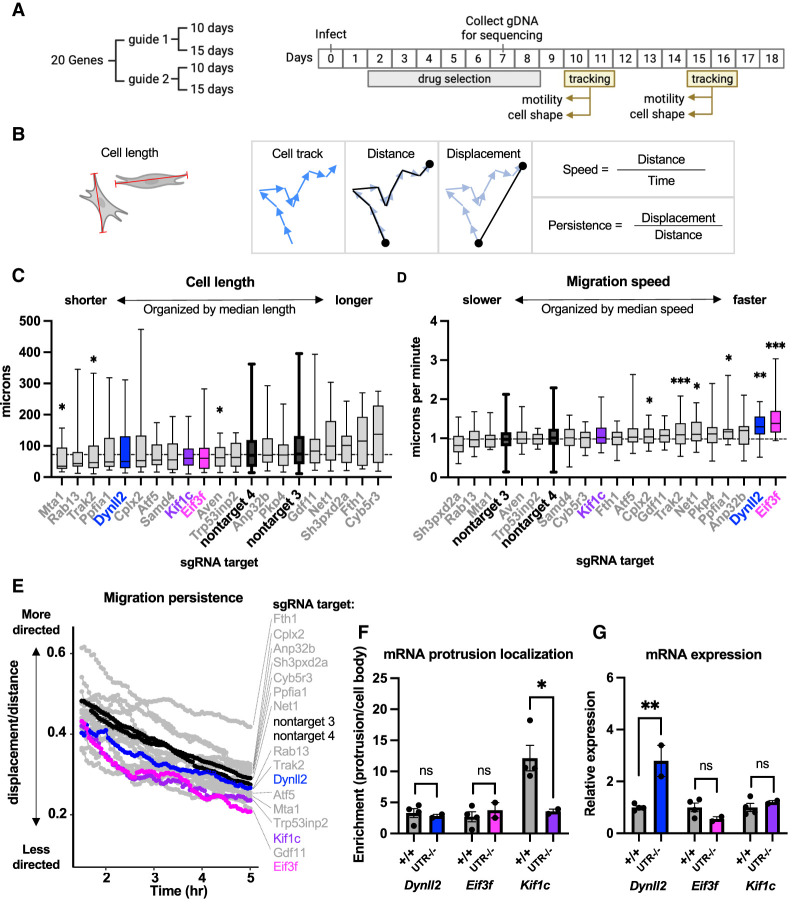
Functional prioritization of localized mRNAs and identification of *Kif1c* as a model for further study. (*A*) Outline of phenotypic screen. Coding sequences of candidate genes were targeted individually with CRISPR/Cas9, selected with puromycin, and then analyzed for phenotypes at two different time points. (*B*) Schematic of phenotypic analyses. (*Left*) Cells were measured along their longest axis (red lines). (*Right*) Speed and persistence were measured from cell tracks (blue arrows) after calculating distance (total path length) and displacement (straight line between first and last time point). (*C*–*E*) Only the top treatment (guide and time point) is shown for each candidate gene. Genes are ordered based on their median measurement; horizontal dashed lines mark the median of nontarget control treatments. Nontarget guides, *Dynll2*, *Eif3f*, and *Kif1c* are denoted in black or color. All other candidates are in gray. Cells were tracked migrating on uncoated plastic dishes for 5 h. *N* = >15 cells per assay for candidate genes. *N* = >150 cells per assay for nontarget guides. (*C*) Box plots of cell length measured from still frames. (*D*) Box plots of migration speed measurements. In *C* and *D*, the box shows the 25th to 75th percentiles, and the line in the middle is the median. Whiskers are drawn down to the smallest value and up to the largest value. (*E*) Migratory persistence over time. (*F*,*G*) Mean and SEM from qRT-PCR on cells with endogenous 3′ UTR deletions. qRT-PCRs on fractionated cells were normalized to the diffuse mRNA *Arpc3*. qRT-PCR on bulk mRNA was normalized to *Ywhaz. N* = 2–4 biological replicates per condition. All statistical tests are unpaired *t*-test compared with +/+. (***) *P* < 0.001, (**) *P* < 0.01, (*) *P* < 0.05.

To identify candidates whose localization was most likely to regulate an observable phenotype in YUMM1.7 cells, we initially used gene loss of function as a proxy. This strategy assumed that a gene with a detectable loss-of-function phenotype would be more likely to manifest a phenotype upon mislocalization of the encoded mRNA, although the severity or nature of the phenotype might be different. The coding sequence of each candidate gene from phase 2 was targeted using lentivirally delivered CRISPR/Cas9 components, resulting in pools of cells containing heterogenous loss-of-function mutations. For each gene, we tested two separate guides, and efficacy of targeting was determined using high-throughput amplicon sequencing. In most cases, both guides for a given gene resulted in a >80% frequency of indels at the target site. Cell pools were also made with nontargeting guides to use as controls in all phenotypic assays. After targeting, cell pools were systematically screened for phenotypic changes in cell shape or cell motility using live-cell tracking (Fig. 2A,B). The tracking assays were performed at two separate time points (10 and 15 d after infection) to account for differences in cellular viability and/or protein stability. Thus, at the completion of the assays, each gene had been analyzed four times (two guides × two time points). For each gene, we selected the single treatment (guide and time point) with the strongest phenotype to be plotted as a representative (Fig. 2C–E). We first measured cell length and observed significantly shorter cells when *Mta1*, *Trak2*, or *Aven* was targeted (Fig. 2C). To quantify cell motility, we used two metrics: speed and persistence (how straight the cell migrates) (Fig. 2B). Cells in which *Dynll2* or *Eif3f* had been targeted migrated the fastest (Fig. 2D), while cells targeted for *Kif1c, Gdf11*, and *Eif3f* migrated the least straight (Fig. 2E). We selected *Kif1c* and *Eif3f* for further analysis because they exhibited strong migration phenotypes and were among the top 10 most localized mRNAs in YUMM1.7 cells. We also selected *Dynll2*, which was among the top 20 most localized mRNAs in YUMM1.7 cells and was associated with a robust increased migration speed phenotype.

#### Phase 4: *cis*-element identification

mRNA localization elements are commonly but not always found in 3′ UTRs. To test whether the 3′ UTR harbors the localization element of *Dynll2*, *Eif3f*, and *Kif1c*, we simultaneously introduced Cas9 and two sgRNAs (one targeting just after the stop codon and one targeting just prior to the polyadenylation sequence) and isolated clonal populations of cells with homozygous 3′ UTR deletions. Loss of either the *Dynll2* 3′ UTR or the *Eif3f* 3′ UTR did not have a significant effect on mRNA localization, suggesting that the *cis*-element is elsewhere in these mRNAs (Fig. 2F), although loss of the *Dynll2* 3′ UTR did result in increased mRNA expression (Fig. 2G). In contrast, loss of the *Kif1c* 3′ UTR abrogated mRNA localization without affecting mRNA expression, making it the best overall candidate for mechanistic study (Fig. 2F,G).

### *Kif1c* mRNA localization requires a short GA-rich element in the proximal 3′ UTR

**Figure 3:**
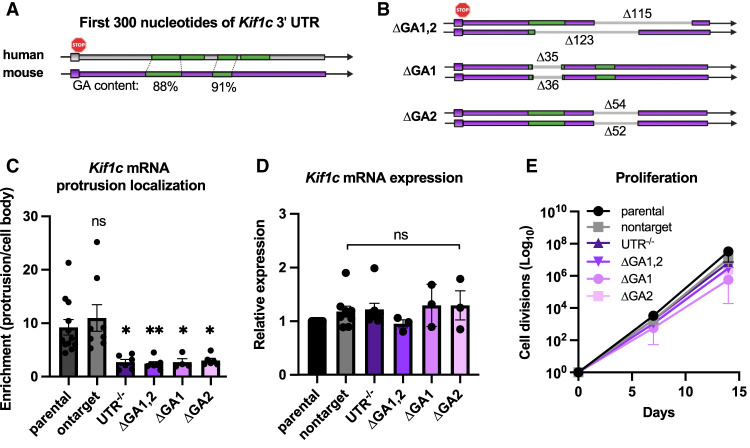
The *Kif1c* mRNA localization element is made up of multiple GA-rich sequences. (*A*,*B*) Schematics of the first 300 nt of the *Kif1c* 3′ UTR. GA-rich elements (green) are highly conserved between mice and humans. (*B*) Schematic of endogenous deletions generated in this study. Unique deletions on each allele of the various ΔGA clonal cell lines are shown. (*C*,*D*) qRT-PCR of *Kif1c* mRNA in cells with endogenous deletions. All qRT-PCR data were normalized to *Ppia* and *Ywhaz*, which were validated to be stably expressed and uniformly distributed in all genetic contexts under study. All statistical tests are ordinary one-way ANOVA compared with parental. *N* = 3–9 biological replicates per condition. (**) *P* < 0.01, (*) *P* < 0.05. (*E*) Cell divisions measured every 2–3 d for 2 wk. *N* = 2–6 biological replicates per condition. Mean + SEM is shown for *C*–*E*.

The *Kif1c* 3′ UTR is >3000 nt long, so we next sought to identify minimal deletions that impair mRNA localization. A previous study using a reporter construct and the *Kif1c* 3′ UTR suggested that GA regions at the proximal end of the 3′ UTR may be most critical (Costa et al. 2020). To test this, we used dual sgRNAs to delete larger (∼130-nt) and smaller (∼35- to 55-nt) portions of the GA elements in the endogenous *Kif1c* 3′ UTR (Fig. 3A,B). Cellular fractionation confirmed that, like deletion of the entire *Kif1c* 3′ UTR, deletion of the GA elements resulted in mislocalization of *Kif1c* mRNA (Fig. 3C). None of the deletions affected total *Kif1c* mRNA abundance or cell viability, except for a modest growth deficiency in *Kif1c*^*ΔGA1*^, which is not uncommon in clonally derived cell lines (Fig. 3D,E). We conclude that *Kif1c*, like *Net1* and *Rab13*, uses GA-rich elements for protrusion localization.

### *Kif1c* mRNA localization does not regulate KIF1C protein abundance or distribution

**Figure 4:**
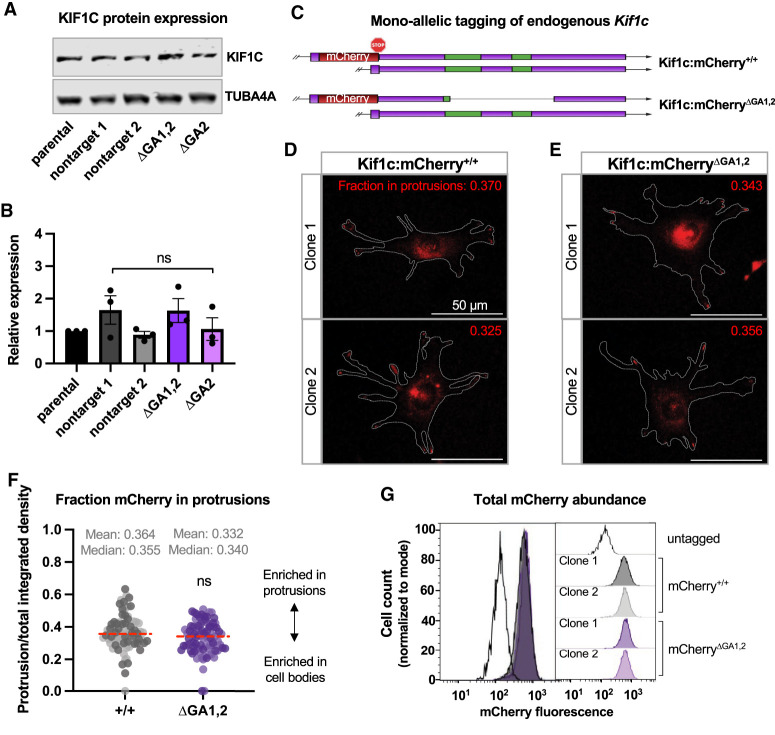
*Kif1c* mRNA localization does not affect KIF1C protein abundance or distribution. (*A*) Representative Western blot of KIF1C in total cell lysate. (*B*) Quantification of KIF1C abundance as measured by Western blotting and normalized to TUBA4A. *N* = 3 biological replicates, showing mean and SEM. (*C*) Schematic of mCherry insertion into the endogenous *Kif1c* locus and subsequent deletion of the ΔGA1,2 element. ΔGA1,2 clones 1 and 2 have 123 and 164 nt deleted, respectively. (*D*,*E*) Representative images of live cells expressing KIF1C:mCherry from localized mRNA (*D*) or mislocalized mRNA (*E*). An outline of the cells is provided in gray. The *inset* number is the value shown in *F* for the imaged cell. (*F*) mCherry fluorescence present in protrusions quantified from cells represented in *D* and *E*. Separate clones for each genotype are represented as lighter and darker points. The experiment was repeated three times with ≥10 cells per clone per experiment. Red dashed line is the median. (*G*) Quantification of total cellular mCherry fluorescence using flow cytometry. More than 12,000 cells per population were used. The same data are shown as an overlay (*left*) and individual populations (*right*). Statistical test in *B* is ordinary one-way ANOVA compared with parental, and statistical test in *F* is unpaired *t*-test.

mRNA localization can be an efficient way to regulate protein abundance and/or distribution. To test whether *Kif1c* mRNA localization affects these parameters, we first isolated protein from various *Kif1c*^+/+^ or *Kif1c*^*ΔGA*^ cell lines. No change in protein abundance, as measured by Western blotting, was detected (Fig. 4A,B). To visualize KIF1C spatially within the cell, we used homologous recombination to tag the C terminus of endogenous KIF1C with mCherry (Fig. 4C). Endogenous KIF1C:mCherry signal was localized perinuclearly and in cell protrusions, as previously described (Fig. 4D; Theisen et al. 2012). Next, we used CRISPR/Cas9 with dual sgRNAs to delete the GA1,2 localization element specifically on the mCherry-tagged allele. Localization of KIF1C:mCherry^ΔGA1,2^ was indistinguishable from KIF1C:mCherry^+/+^ and was readily found in cell protrusions (Fig. 4D–F). Finally, we used flow cytometry to measure changes in mCherry abundance. In agreement with our Western blot data, no change in protein abundance was observed (Fig. 4G). Thus, *Kif1c* mRNA localization does not detectably regulate KIF1C protein abundance or subcellular distribution.

### *Kif1c* mRNA localization is required for directional cell migration but is dispensable for APC-dependent mRNA trafficking

**Figure 5:**
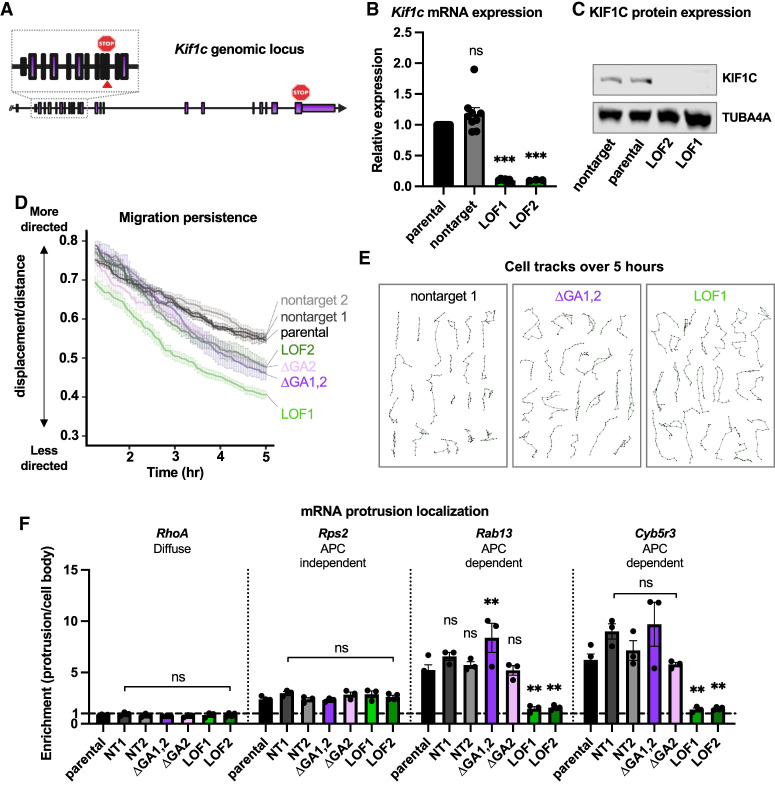
*Kif1c* mRNA localization is required for directed cell migration but dispensable for mRNA trafficking. (*A*) Schematic of the *Kif1c* genomic locus. The red arrowhead and stop sign in the *inset* denote cut site and location of premature termination codon in LOF alleles, respectively. LOF1 and LOF2 harbor 2- and 4-bp deletions in exon 11, respectively. (*B*) qRT-PCR on cells with endogenous deletions; mean and SEM are shown. *N* = 3–9 biological replicates. (*C*) Representative Western blot of total protein lysates. The experiment was repeated three times. (*D*,*E*) Cells were plated sparsely on fibronectin-coated plastic dishes for 18–24 h and then tracked every 3 min for at least 5 h. (*D*) Plot of migration persistence over time. Fifteen or more cells per genotype were used per experiment. The experiment was repeated three or more times per genotype. Error bars show SEM. (*E*) Individual cell tracks from a single replicate for the given genotypes. (*F*) qRT-PCR measurement of mRNA enrichment in protrusions in the indicated cell lines; mean and SEM are shown. All qRT-PCR data were normalized to *Ppia* and *Ywhaz*. *N* = 3–4 biological replicates per condition. All statistical tests are ordinary one-way ANOVA compared with parental. (***) *P* < 0.001, (**) *P* < 0.01.

Knockdown of KIF1C has been independently shown to cause defects in directed cell migration and mRNA trafficking (Theisen et al. 2012; Pichon et al. 2021). Whether these processes are interdependent is unclear. Likewise, a causal relationship between *Kif1c* mRNA localization and downstream KIF1C functions has not been established. We hypothesized that mislocalization of *Kif1c* mRNA would lead to defects in KIF1C function and result in cell migration and mRNA trafficking defects. To test this, we first generated clonal loss-of-function mutants (*Kif1c*^*LOF*^) to serve as positive controls in our assays. To this end, we targeted the *Kif1c* coding sequence with CRISPR/Cas9 and obtained clonal *Kif1c*^*LOF*^ cells with biallelic early frameshift mutations, which introduce premature stop codons. As expected, *Kif1c*^*LOF*^ mutant cells exhibited decreased *Kif1c* mRNA expression and no detectable protein (Fig. 5A–C).

We first measured the migration persistence of *Kif1c*^+/+^, *Kif1c*^*ΔGA*^, and *Kif1c*^*LOF*^ cells (as depicted in Fig. 2B), a metric of directed cell migration. Cells were plated sparsely on fibronectin-coated plates, allowed to acclimate overnight, and then tracked for 5 h. As expected, *Kif1c*^*LOF*^ cells did not migrate as persistently as *Kif1c*^+/+^ cells. Consistent with our hypothesis that mRNA localization is required for KIF1C function, *Kif1c*^*ΔGA*^ cells resembled *Kif1c*^*LOF*^ cells and displayed diminished directed cell migration (Fig. 5D,E).

We next measured mRNA trafficking by fractionating cells and examining the localization of multiple protrusion-enriched mRNAs. As expected, the APC-dependent mRNAs *Rab13* and *Cyb5r3* did not localize properly in *Kif1c*^*LOF*^ cells, while the APC-independent mRNA, *Rps2*, localized normally. Surprisingly, the APC-dependent mRNAs *Rab13* and *Cyb5r3* were properly localized in *Kif1c*^*ΔGA*^ cells (Fig. 5F). Thus, in contrast to the prediction that *Kif1c* mRNA localization is required for APC-dependent mRNA trafficking, we observed that KIF1C's role in mRNA trafficking does not require *Kif1c* mRNA to itself be localized. Taken together, these results demonstrate that the functions of KIF1C in cell migration and mRNA trafficking are separable and suggest that there are multiple pools of KIF1C protein that are established through localization of the *Kif1c* mRNA. Furthermore, the observation that mRNA trafficking occurred properly in *Kif1c*^*ΔGA*^ cells is consistent with our observation that these cells still have KIF1C in their protrusions, the destination of KIF1C when trafficking other mRNAs (Fig. 4C–E).

### Identification of endogenous KIF1C-interacting proteins

These data established that *Kif1c* mRNA localization is required for proper cell migration. However, as mRNA localization did not affect the distribution or abundance of KIF1C protein, the question remained as to how mRNA localization regulates KIF1C function. Like other kinesins, KIF1C carries cargoes along microtubules and can modulate different cellular processes dependent on which cargo it carries. We hypothesized that changes in mRNA localization may affect KIF1C function by impacting its binding partners, since mRNAs in the cell body or protrusions would be translated in the context of distinct sets of potential interactors of the nascent protein. To test this hypothesis, we carried out immunoprecipitation (IP) of endogenous KIF1C from *Kif1c*^+/+^ and *Kif1c*^*ΔGA1,2*^ cells, with *Kif1c*^*LOF*^ cells included as a negative control, followed by mass spectrometry to identify interacting proteins (Supplemental Table S2). We chose to immunoprecipitate endogenous KIF1C because overexpression of KIF1C leads to cellular phenotypes, such as increased Golgi object size (Lee et al. 2015) and length of cell tails (Theisen et al. 2012), and we did not want to overwhelm any regulatory processes acting on the protein.

**Figure 6:**
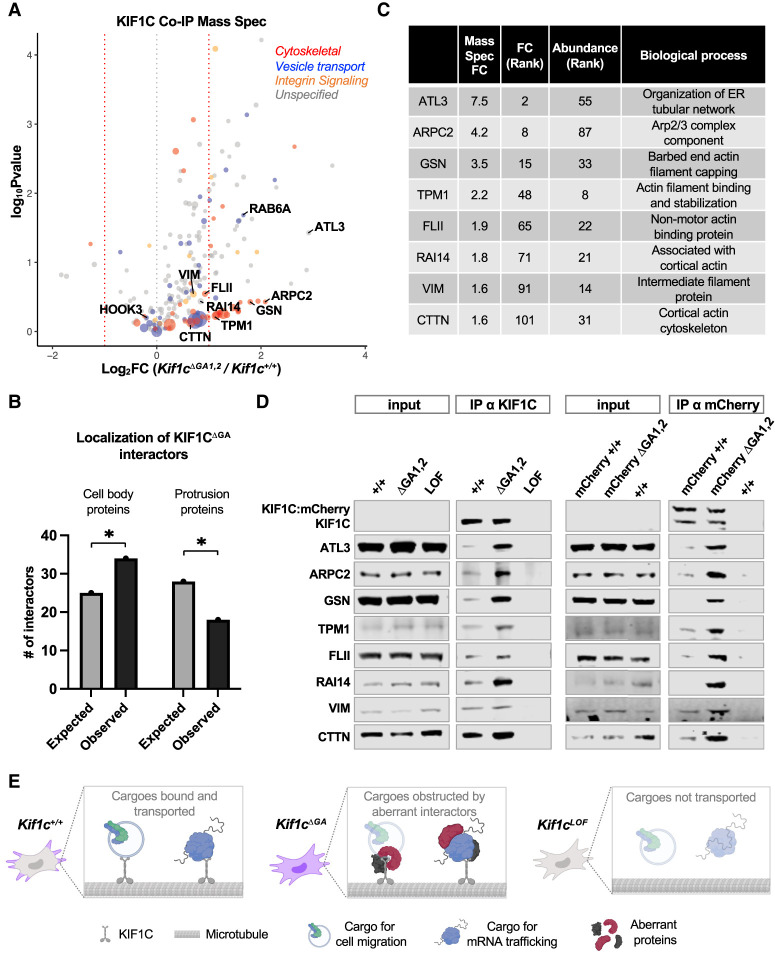
Identification of endogenous KIF1C binding partners and their regulation by *Kif1c* mRNA localization. (*A*) Mass spectrometry of endogenous KIF1C IPs; the size of the dot represents the abundance of the interactor. Only hits more than threefold enriched over *Kif1c*^*LOF*^ were included. Functional categories were determined using PANTHER and manual curation; many genes fall into more than one category. *N* = 3 biological replicates per condition. (*B*) Analysis of the expected and observed numbers of cell body- and protrusion-enriched proteins among the KIF1C^ΔGA^-preferred interactors (interactors with log_2_FC > 0). (*) *P* < 0.05 (χ^2^). (*C*) Fold change (FC; *Kif1c*^*ΔGA*^*/Kif1c*^+/+^), abundance rank, and a brief description of candidate proteins selected for validation. (*D*) Western blot analysis of co-IP assays with endogenous KIF1C from untagged cells (*left*) or mCherry-tagged KIF1C cells (*right*) using α-KIF1C or α-mCherry antibodies, respectively. Note that endogenous KIF1C is expressed at low levels and is undetectable in input samples under these conditions. (*E*) Proposed model: In *Kif1c*^+/+^ cells, *Kif1c* mRNA (purple) localizes to protrusions. KIF1C protein undergoes limited, specific protein–protein interactions and transports cargoes related to mRNA trafficking (e.g., APC) and cell migration (e.g., integrin). In *Kif1c*^*ΔGA*^ cells, *Kif1c* mRNA is diffuse in the cell body, and KIF1C protein engages with additional, aberrant interactors. KIF1C is still able to transport cargoes for mRNA trafficking, but cargoes for cell migration are affected through an unknown mechanism (faded cell migration cargo), which may include the cargo being lost, diminished, or unable to recycle properly. In *Kif1c*^*LOF*^ cells, *Kif1c* mRNA and KIF1C protein are absent, and all cargos are affected.

Among the interactors detected in *Kif1c*^+/+^, *Kif1c*^*ΔGA1,2*^, or both cell lines, we observed known KIF1C binding partners such as RAB6A and HOOK3 (Fig. 6A; Lee et al. 2015; Siddiqui et al. 2019). A substantial number of the interactors (>40%) were related to the cytoskeleton, integrin signaling, and/or vesicle transport (colored dots in Fig. 6A). We looked for overrepresentation of biological pathways by comparing the interacting proteins with all genes expressed in YUMM1.7 cells. The broad classes of integrin signaling and cytoskeletal proteins were indeed enriched (5.8-fold and 6.5-fold, respectively). More striking, however, was the overrepresentation of barbed-end actin filament-capping proteins (17-fold) and Arp2/3 complex proteins (26.5-fold). In other cell types, KIF1C is thought to provide an interface for actin and tubulin, which is required for the formation of actin-based invasive protrusions called podosomes. Whether KIF1C is also serving in this role in melanoma cells or interfaces with actin to serve another role remains to be determined.

### Loss of *Kif1c* mRNA localization leads to dysregulated protein–protein interactions

When we plotted the relative change in binding interactions (*Kif1c*^*ΔGA1,2*^/*Kif1c*^+/+^), we noticed a pronounced skew of the volcano plot toward the right, suggesting that most interactions were increased when KIF1C was produced from mislocalized mRNA (Fig. 6A). One interpretation of this skew is that proper mRNA localization allows for preferential loading of selected KIF1C interactors that may be coenriched in protrusions, while KIF1C produced from mislocalized mRNA is loaded more promiscuously in the cell body. A prediction of this model is that the aberrant interactors in *Kif1c*^*ΔGA*^ cells would be proteins generally enriched in the cell body compared with protrusions. We tested this prediction by querying our interactors against a previously generated data set that categorized proteins as being significantly enriched in cell bodies or protrusions (Dermit et al. 2020). Consistent with our model, interactors of KIF1C produced from mislocalized mRNA were enriched for cell body-specific proteins, while protrusion-specific proteins were relatively depleted (Fig. 6B).

To validate these results, we ranked hits based on their absolute abundance in the mass spectrometry data and their fold change of enrichment in *Kif1c*^*ΔGA1,2*^ compared with *Kif1c*^+/+^ cells. From this group, we selected eight proteins that represent a range of differential enrichment values and included a barbed-end actin filament-capping protein (GSN) and an Arp2/3 complex protein (ARPC2) (Fig. 6C). Co-IP assays using an α-KIF1C antibody followed by Western blotting confirmed that these protein–protein interactions were increased upon *Kif1c* mRNA mislocalization (Fig. 6D). As an independent approach, we also carried out co-IPs using an α-mCherry antibody in the KIF1C:mCherry-tagged cell lines and observed the same results (Fig. 6D). Because only one allele of *Kif1c* was tagged, the α-mCherry co-IPs also allowed us to distinguish nontagged KIF1C, which copurified with tagged KIF1C due to dimerization irrespective of mRNA localization. In both untagged and tagged cell lines, we observed a reproducible range of enrichment of the validated KIF1C interactors. For example, some proteins (e.g., VIM and CTTN) were modestly enriched in *Kif1c*^*ΔGA1,2*^ cells yet still readily detectable in *Kif1c*^+/+^ cells, while other proteins (e.g., GSN and ATL3) were barely detectable in *Kif1c*^+/+^ cells. We speculate that the former class of proteins may represent normal interaction partners that become dysregulated in response to mRNA mislocalization, while the latter class may be aberrant interactors that opportunistically bind KIF1C when the mRNA is not translated in the proper context. In conclusion, these data reveal a critical role for *Kif1c* mRNA localization in dictating the specificity of KIF1C protein–protein interactions.

## Discussion

Subcellular mRNA localization is a widely used mechanism for increasing protein enrichment at a given site, such as in neurites or organelles (Engel et al. 2020; Gasparski et al. 2022). In contrast, mRNA localization to protrusions in nonneuronal cells does not give rise to protrusion-enriched proteins, yet is absolutely required for proper cell migration. Thus, mRNA localization to protrusions appears to serve a noncanonical and poorly understood purpose. Here, we demonstrate that *Kif1c* mRNA localization to protrusions is required for establishing the specificity of protein–protein interactions independently of protein abundance or localization. Like other APC-dependent mRNAs, the *Kif1c* 3′ UTR contains multiple GA-rich regions. While the precise identity of the minimal localization element remains unknown, we demonstrate that both GA-rich elements in the proximal *Kif1c* 3′ UTR are required, as deletion of either decreases the ability of the mRNA to localize. Furthermore, we show that KIF1C protein function is predicated, at least in part, on localization of *Kif1c* mRNA, and that KIF1C's downstream roles in cell migration and mRNA trafficking are mechanistically separable. Recently, it was shown that localization of another APC-dependent mRNA, *Rab13*, is required for proper loading of a key interaction partner (Moissoglu et al. 2020). The fact that *Rab13* and *Kif1c* are among the first APC-dependent mRNAs to be studied in detail strongly suggests that the regulation of binding partner specificity by mRNA localization will be a common mechanism in nonneuronal cells. Furthermore, we predict that RAB13 interactions may be more broadly dysregulated than previously appreciated.

These findings expand our understanding of how sequence elements in 3′ UTRs can regulate protein–protein interactions. In addition to serving as localization elements, sequences in 3′ UTRs can recruit specific proteins that are then poised to interact with the nascent polypeptide as it is translated, thus impacting the subsequent subcellular localization and function of the encoded protein (Berkovits and Mayr 2015). Altogether, these findings illustrate the diversity of mechanisms through which sequence elements in 3′ UTRs can govern protein function independently of regulatory effects on mRNA abundance or translation.

Our data show that *Kif1c* mRNA localization regulates cell migration but not mRNA trafficking. We hypothesize that these differential sensitivities to mRNA localization could, in some contexts, provide a mechanism for regulating KIF1C activity. For example, increased or decreased localization of the *Kif1c* mRNA would provide a means to divert the pool of KIF1C in order to enhance or suppress migration, as opposed to simply tuning the overall level or activity of the protein, which would also affect mRNA trafficking. Furthermore, because this would provide a post-transcriptional mechanism for regulating cellular behavior, it would allow for rapid responses to extracellular cues (Chrisafis et al. 2020; Moriarty et al. 2022). Examination of whether and how the degree of *Kif1c* mRNA localization is regulated is therefore an interesting direction for future investigation.

How *Kif1c* mRNA localization to protrusions modulates protein–protein interactions is unclear. One possibility is that mRNA localization permits only key interaction partners to gain privileged access to nascent proteins in an otherwise crowded cellular milieu. Without sequestration, ubiquitous cellular proteins may outcompete proper interaction partners for binding (see model in Fig. 6E). The variable effect of mRNA localization on downstream functions may derive from differential sensitivity to changes in binding partners. For example, cell migration requires continual recycling of integrins and is known to be sensitive even to overexpression of wild-type KIF1C protein (Theisen et al. 2012). As such, subtle changes in binding specificity may be sufficient to inhibit this process. Conversely, while less is known about mRNA trafficking, based on our results, we predict the interactions that regulate this process will be comparatively more stable and/or robust. Future work will need to address how individual interactors are differentially recruited and loaded and which are causal for specific phenotypes. We predict that such studies not only will explain the many roles of *Kif1c*, but will also improve our understanding of the functional significance of many other protrusion-localized mRNAs present across nonneuronal cell types.

## Materials and methods

### Cell culture

Established YUMM1.7 and COS1 cell lines were obtained from ATCC. COS1 cells were cultured in DMEM supplemented with 10% FBS and 5% penicillin/streptomycin. YUMM1.7 cells were cultured in DMEM:F12, HEPES with L-glutamine supplemented with 10% FBS, 5% nonessential amino acids, and 5% penicillin/streptomycin. The YUMM1.7 cell line was authenticated after receipt using allele-specific primers and confirmed to be negative for mycoplasma.

### Fractionation of cells, RNA-seq, and qRT-PCR

PET Millicell hanging cell culture inserts with 1-μm pores sized for six-well plates were coated on the bottom side with 30 ug/mL fibronectin. Fibronectin was diluted in sterile PBS, and inserts were coated for 15 min, aspirated, and allowed to dry. Cells were plated at 4 × 10^5^ to 8 × 10^5^ per membrane and placed in an incubator overnight. For fractionation, media was aspirated, cells were rinsed with RNase-free PBS and aspirated, and then cell bodies were scraped using a cell scraper and collected into 600 μL of RLT buffer from the Qiagen RNeasy kit. Inserts were rinsed with RNase-free PBS and cleaned with a cotton swab three times. The cleaned insert was carefully dislodged, placed inside a new tube with 600 μL of RLT buffer, and briefly vortexed. After cell bodies and protrusions were collected into RLT for each sample, the standard protocol for RNeasy was followed, including DNase digestion. RNA was eluted with 30 μL of RNase-free water. Sixteen microliters of protrusion RNA or 1 μL of cell body RNA was used for making cDNA with Takara PrimeScript RT master mix. cDNA was diluted 1:10, and 4 μL was used to carry out qPCR with power SYBR reagents. For qPCR, one to three inserts were used per sample. For RNA-seq, samples were prepared as above except samples were collected into Qiazol, and the Qiagen miRNeasy kit was used to allow for capture of small RNAs. There were two RNA-seq replicates, each with six inserts and 6 × 10^5^ to 8 × 10^5^ cells per insert. Libraries were generated from total RNA using TruSeq stranded total RNA LT sample preparation kit from Illumina and depleted for rRNA. Samples were run on the Illumina NextSeq 500 using V2.5 reagents and subjected to strand-specific, single-read, whole-transcriptome sequencing at a depth of 25 million to 35 million reads per sample. Quality assessment of the reads was done using FastQC. Reads were aligned to mouse reference genome mm10 using TopHat (v2.0.12), and differential expression analysis was carried out using edgeR. For qRT-PCR on bulk cellular RNA, RNA was prepared using RNeasy and then quantified, and an equal amount from each sample was used to make cDNA; cDNA was diluted 1:20 before use. For the candidate screen (Fig. 2), fractionated qRT-PCR data were first normalized to *Arpc3*, which is uniformly localized, and then scaled so that the localized gene *Pkp4* was equal in each experiment. This compensated for variability in the fractionation experiments. For all following qRT-PCR experiments (including bulk or fractionationed mRNA), qRT-PCR data were only normalized to the geometric mean of the uniformly distributed *Ppia* and *Ywhaz* mRNAs, and fractionation values were not scaled. *Ppia* and *Ywhaz* were confirmed to be stably expressed using NormFinder (Andersen et al. 2004). All primer sequences are provided in Supplemental Table S3.

### Protrusion enrichment across cell types

RNA localization data sets were downloaded from their original sources. One source was used for NIH3T3 cells (Wang et al. 2017), and two sources were used for MDA-MB-231 cells (Jakobsen et al. 2013; Mardakheh et al. 2015). All data sets were processed in Orthoretriever to convert gene IDs to gene names and to convert human genes to their mouse ortholog. The MDA-MB-231 data sets were merged such that, for any given gene, the higher measured protrusion enrichment value was maintained.

### Lentivirus production, generation of pooled knockout populations, and amplicon sequencing

Heterogenous knockout pools were generated using lentiCRISPR_v2 as described previously (Golden et al. 2017) using the top two guides for each gene from the Brie sgRNA library (Doench et al. 2016). Briefly, guides for candidate genes or two nontargeting guides were cloned into lentiCRISPR_v2 (Addgene 52961). Virus was generated by plating 3.5 × 10^4^ COS1 cells per well in a six-well plate and transfecting them on the second day with 1 μg of total plasmid per well at a 5:3:2 ratio of lentiCRISPR:psPAX2 (Addgene 12260):pMD2.G (Addgene 12259) using FuGENE HD. Viral media was collected after 48 and 72 h, passed through a 0.45-mm filter, and stored as aliquots at −80°C. Viral media was diluted 1:1 with YUMM1.7 complete media including 8 μg/mL polybrene and used to transduce YUMM1.7 cells in 12-well plates. Media was changed 24 h after transduction, and 2 μg/mL puromycin selection was started after an additional 24 h. Cells were selected for 6 d and expanded into 10-cm plates. gDNA for sequencing was collected on selection day 5 and prepared using Qiagen DNeasy. Sequencing libraries were prepared through two rounds of PCR: (1) gene-specific primers with overhangs and (2) overhang-specific primers for indexing and multiplexing. PCRs from each sample were concentration-matched, pooled, run on a 2% agarose gel stained with SybrSafe, gel-purified with Qiagen gel extraction kit, and sequenced on a MiSeq using MiSeq reagent Nano kit v2 at a depth of 1 million reads. Resulting FastQ files were analyzed using CRISPResso (Clement et al. 2019). Cells were plated for live-cell tracking on days 10 and 15 after infection, (which were days 8 and 13 after selection, respectively) and counted for proliferation every 2–3 d throughout. The phenotypic screen was carried out in rounds. Each round included two nontarget guides (except for the first round, which only included nontarget 3), as well as guides for two to four candidates. For statistical analyses shown in Figure 2, candidate phenotypes were compared with nontarget 3 phenotypes measured from the same round. However, for graphing purposes in Figure 2, nontarget measurements from all rounds of analyses were plotted together. Targeting *Cenpb* generated only ∼10% indels, and these cells were not included in the representative phenotypic analyses.

### Live-cell tracking

Live-cell tracking of single cells was carried out in 24-well dishes on a heated stage with atmospheric control using a Zeiss AxioObserver Z1 equipped to automatically perform time-lapse live-cell image acquisition. Cells were plated dilutely (∼500 cells per well) in plastic dishes without (Fig. 2) or with (Fig. 5) fibronectin coating, allowed to acclimate overnight, and then imaged using phase contrast and a 10× EC PlnN air objective with NA = 0.3 every 3 min for 8 h. Cells were manually tracked using Trackmate in Fiji. Cells that were in view over a 5 h period were used for analysis. For tracking on fibronectin, cells were excluded if they divided during the tracking window. Tracking data were processed with the chemotaxis tool (Ibidi) and then analyzed using Rstudio with a code prepared in-house. The code performed the following analyses: (1) For every cell, the distance and displacement were recorded for every frame. Distance is not “direction aware” and is the cumulative movement of the cell, also called the total path length. Displacement is “direction aware” and is the straight line distance from the original position of the cell to the final position of the cell. (2) For every cell, the persistence at every frame was calculated (persistence = displacement/distance). (3) For every frame, the mean persistence across all cells of a given genotype was calculated. Only cells that migrated for 100 frames were analyzed. For experiments with replicates, the mean of the replicates and the standard error of the mean were plotted for each time point. Speed was calculated as the total distance divided by the total time for individual cells, and only cells that were visible in ≥60 consecutive frames were analyzed. Cell shape was analyzed by measuring cell features in Fiji of all cells in view during the 50th frame of every movie. Cell length is the longest straight line that can be drawn for a given cell from tip to tip. Cell tracks were generated using DiPer (Gorelik and Gautreau 2014).

### Generation of clonal cell lines with deletions and insertions

To generate clonal cell lines, cells were transfected with px458 (Addgene 48138), which expresses GFP, Cas9, and sgRNA. Guides were selected using the UCSC genome browser-NGG target site function. GFP^+^ cells were single-cell-sorted 48 h after transfection. Clones were expanded and genotyped using primers spanning sgRNA cut sites. For deletions, two sgRNA were used; for indels, one sgRNA was used. The genotype of mutated alleles was determined using Sanger sequencing. For mCherry insertion via homologous recombination, one sgRNA targeting just after the stop codon was used, and px458 was cotransfected with a plasmid containing linker-mCherry flanked by >1000-nt *Kif1c* homology arms in which the PAM site for the sgRNA was altered to inhibit recutting after repair. Transfected cells were first sorted as GFP^+^ pools after 48 h and then single-cell-sorted for mCherry^+^ cells 6 d later. The tagged and untagged alleles were analyzed using Sanger sequencing. sgRNA sequences are in Supplemental Table S3.

### Live-cell fluorescent imaging and analysis

Live-cell imaging of fluorescent cells was carried out in Nunc Lab-Tek II eight-well chambered coverglass in live-cell imaging solution (Invitrogen) using a 40× EC PlnN oil objective with NA = 1.3 on a Zeiss AxioObserver Z1 with an AxioCam MRm monochrome digital camera. Coverglasses were coated with 30 μg/mL fibronectin diluted in PBS for 15 min, aspirated, and allowed to dry. mCherry-tagged cells and an untagged parental cell line were plated dilutely and allowed to adhere for 2 h. Cells were rinsed and then imaged in prewarmed imaging media. Six slices, 0.7 μm apart, with 1500-msec exposure time were captured for each cell using an HXP 120C light source and filter set 63HE, as well as a bright-field image. Cells were analyzed using Fiji as follows: Cell outlines were manually drawn. Cell bodies versus protrusions were distinguished using white light. The cell body was defined by edge of the rough matter surrounding the nucleus, which coincided with flexion points in the cell membrane near protrusions. Only the slice in which the base of the cell was sharply in focus was quantified. Only cells that unambiguously did not overlap with other cells or debris were used. Protrusion enrichment was calculated as follows: (1) The mean fluorescence per unit area for protrusions and cell bodies was calculated for every cell, including negative controls. (a) The integrated density and area of the whole cell and the cell body were measured. (b) Protrusion integrated density = whole cell integrated density − cell body integrated density. Protrusion area = whole cell area − cell body area. (c) Mean fluorescence (cell body or protrusions) = integrated density/area. (2) The average mean fluorescence in protrusions and cell bodies due to background autofluorescence was calculated by averaging the measurements from >10 negative control cells for each experiment. (3) The average protrusion-specific and cell body-specific background (quantified in step 2) was subtracted from protrusions and cell bodies of tagged cells. (4) The corrected protrusion and cell body fluorescence values were used to calculate fluorescence in protrusions/cell bodies.

Whole-cell fluorescence of tagged cell lines was measured using a BD Melody FACS and FlowJo software. Twelve-thousand to 16,000 tagged single cells per cell line were measured using the PE-CF594 laser. Cells were gated to visualize only living, single cells and then plotted with mCherry (height) on the *X*-axis.

### Coimmunoprecipitation mass spectrometry and Western blotting

Cells (4 × 10^5^ to 1.5 × 10^6^) were plated 2–4 d before collection in 15-cm dishes such that they were 90% confluent on the day of collection, with two dishes per sample. Samples were prepared in the cold room by rinsing briefly with ice-cold PBS, aspirating the PBS, and scraping the cells into a 1.5-mL tube using residual PBS. Cells were immediately spun at 1000*g* for 1 min and aspirated, and 300 μL of Thermo cell lysis buffer (NN0011) with 2× complete protease inhibitor and 2× PhosSTOP was added. Lysates were resuspended with gentle pipetting, rotated for at least 30 min at 4°C, and pelleted at 14,000*g* for 10 min, and supernatants were moved to a new tube. Lysates were precleared with 50 μL of washed protein G Dynabeads for 30 min, beads were discarded, lysate concentration was measured using Pierce BCA protein assay kit, and the volume and concentrations for each genotype (e.g., +/+, ΔGA, and LOF) were matched using extra lysis buffer as needed. For co-IP mass spectrometry, 4.5–5 mg of total protein was used for each replicate with 4 μg of KIF1C antibody (Kif1c/LTXS1; Bethyl Laboratories A301-072A). For co-IP Western blots, 3–4 mg of total protein and 2 μg of KIF1C antibody or 10 μg of mCherry antibody (mCherry monoclonal antibody; Invitrogen 16D7) were used. Antibody was added directly to lysates, the mixture was rotated for 90 min at room temperature, and then 75 μL of rinsed protein G Dynabeads was added and rotated for an additional 30 min at room temperature. Unbound proteins were removed by washing three times with wash buffer (50 mM Tris at pH 7.4, 150 mM NaCl, 0.02% NP-40, 1× PhosSTOP, 1× complete protease inhibitor), transferring the beads to a fresh tube, and eluting in 18 μL of 1× Laemmli buffer (Boston Bioproducts BP-111R). All of the eluate was run on NuPAGE 4%–12% Bis-Tris protein gels in 1.5-mm 15-well plates. For mass spectrometry, the proteins were run 10 mm into the gel, labeled with SimplyBlue SafeStain, carefully excised, and submitted for analysis on a Lumos mass spectrometer. Data were analyzed with Proteome Discoverer 2.4 and searched using the mouse protein database from UniProt. Proteins that were identified by a single peptide or identified but not quantified were omitted from analysis.

For Western blotting, whole-cell lysates were collected from confluent cells grown in a six-well plate. Cells were rinsed with ice-cold PBS and then scraped in 80 μL of cold RIPA buffer (50 mM Tris-HCL at pH 7.4, 1% NP-40, 0.5% Na-deoxycholate, 0.1% SDS, 150 mM NaCl) with 1× complete EDTA-free protease inhibitor (Sigma). Lysates were agitated for 20 min at 4°C and pelleted, and the supernatant was transferred to a new tube. Samples were diluted to 1× Laemmili. SDS-PAGE was run on NuPAGE 4%–12% Bis-Tris protein gels until the 20-kDa band of the protein ladder ran off the gel, and then the proteins were transferred to nitrocellulose membranes, blocked with milk, and blotted 1:1000 with the designated antibodies: TPM1/28477-1-AP, ARPC2/15058-1-AP, CTTN/11381-1-AP, GSN/11644-2-AP; RAI14/17507-1-AP, FLII/67039-1-Ig, and ATL3/16921-1-AP from Proteintech; VIM/sc-6260 from Santa Cruz Biotechnology; and TUBA4A/T6199 from Sigma. The following secondaries were used at 1:10,000 dilution: IRDye 800CW donkey antirabbit or antimouse IgG and IRDye 680LT goat antirabbit or antimouse from Li-Cor. For ATL3 and VIM, the following light chain-specific secondary from Fisher was used: IgG fraction monoclonal mouse antirabbit IgG, light chain-specific 790. Blots were imaged on a Li-Cor Odyssey Clx imaging system and analyzed with ImageStudio. For quantification, KIF1C and TUBA4A values were measured using ImageStudio.

### Quantification of protein enrichment in cell bodies and protrusions.

A list of human genes categorized across six cell types as encoding proteins that are significantly protrusion-enriched, significantly cell body-enriched, or not significantly enriched in either location was obtained from Dermit et al. (2020). Gene names were converted to mouse orthologs using Orthoretriever (5619 of 5905 genes successfully converted). The gene list was filtered to include only genes with TPM of >1 in YUMM1.7 cell bodies based on RNA-seq data (4954 genes). For the remaining proteins, the percentage localized in each cellular compartment was calculated (20% of proteins were protrusion-enriched, 18% of proteins were cell body-enriched, and 62% of proteins were not significantly enriched in either compartment). The percentages were used to determine the expected number of interactors from each compartment in the KIF1C mass spectrometry data set.

### Data access

RNA sequencing data generated in this study have been deposited in GEO (accession no. GSE219089).

## Supplementary Material

Supplemental Material
