# Supplementary material for: Localization of Kif1c mRNA to cell protrusions dictates binding partner specificity of the encoded protein
Source: Genes Dev. 2023 Mar 1;37(5-6):191–203. doi: 10.1101/gad.350320.122 (PMC10111864; doi:10.1101/gad.350320.122)
Supplement: Supplemental Material [file supp_37_5-6_191__DC1.html]

Localization of Kif1c mRNA to cell protrusions dictates binding partner specificity of the encoded protein — Localization of Kif1c mRNA to cell protrusions dictates binding partner specificity of the encoded protein — Supplemental Material 

# Localization of *Kif1c* mRNA to cell protrusions dictates binding partner specificity of the encoded protein

## Supplemental Material

- Supplemental\_table\_S1.xlsx
- Supplemental\_table\_S2.xlsx
- Supplemental\_table\_S3.xlsx
- Supplemental\_legends.pdf
