# Supplementary material for: Localization of Kif1c mRNA to cell protrusions dictates binding partner specificity of the encoded protein
Source: Genes Dev. 2023 Mar 1;37(5-6):191–203. doi: 10.1101/gad.350320.122 (PMC10111864; doi:10.1101/gad.350320.122)
Supplement: Supplemental Material [file supp_gad.350320.122_Supplemental_legends.pdf]

SUPPLEMENTAL TABLES

Supplemental Table S1:

- RNA-seq results
- Comparative protrusion enrichment values

Supplemental Table S2:

- Mass spec results

Supplemental Table S3:

- Guide sequences used for Lenticrispr_v2
- Guide sequences used for generating clonal cell lines
- Genotyping primers for analyzing CRISPR alleles
- Primers for qPCR
- Primers for MiSeq
